# Limonene as a Renewable Platform Molecule: Chemical Modifications and Polymerization Strategies toward Advanced Materials

**DOI:** 10.1021/acspolymersau.5c00192

**Published:** 2026-01-23

**Authors:** Mateus Teixeira Bertão, Roniérik Pioli Vieira

**Affiliations:** Universidade Estadual de Campinas (UNICAMP), School of Chemical Engineering (FEQ), Albert Einstein Avenue, 500, Campinas, São Paulo 13083-852, Brazil

**Keywords:** biobased polymers, limonene polymerization, ROCOP, sustainable monomers, circular economy, thiol−ene polymerization, orange peel, essential oil, citrus, agroindustry byproducts

## Abstract

Significant reliance on petroleum-based plastics remains due to their attractive properties and wide-ranging applications. Driven by environmental concerns, recent research has increasingly focused on utilizing naturally occurring plant-derived molecules and environmentally friendly processes for the synthesis of novel polymeric materials with adequate properties to replace petroleum-based materials. Within this context, limonene has gained unusual prominence as an abundant citrus byproduct. This terpene can be functionalized through a variety of classical organic reactions, e.g., epoxidation, (meth)­acrylation, lactam formation, and thiol–ene click chemistry, opening distinct pathways toward structurally diverse polymers. These routes span traditional radical and ionic processes, as well as coordination systems and ring-opening polymerizations. Together, they have enabled materials that range from poly­(limonene carbonates) and semiaromatic polyesters to polyethers, biobased polyamides, thermosets, and photo-cross-linkable resins suitable for 3D and 4D printing. Many of these polymers have demonstrated promising optical, mechanical, or thermal performance, although important challenges persist, particularly regarding dispersity control and the integration of recycling strategies into circular economy cycles. By bringing these developments into a single narrative, this review highlights how limonene is gradually shifting from a fragrance molecule to a versatile precursor for advanced, renewable polymeric materials.

## Graphical abstract

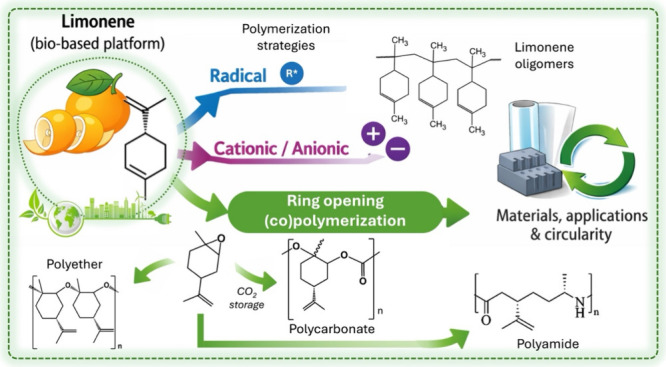

## 1 Introduction

The widespread use of petroleum-based polymers persists due to their versatile properties and essential role in numerous daily life applications. However, their nonbiodegradable nature contributes to the accumulation of at least 14 million tons of plastic waste in landfills and oceans every year.^1^ This scenario has intensified the search for polymeric materials derived from renewable, nonpetroleum resources through chemical routes aligned with the principles of green chemistry. In this context, naturally abundant molecules combined with innovative or optimized processes have emerged as promising strategies to reduce environmental impact and production costs. Growing interest from academia and industry reflects not only the urgency of sustainable development but also concerns regarding the progressive depletion of petroleum reserves.^1–4^

Among renewable feedstocks, natural compounds such as vegetable oils, polysaccharides (e.g., starch and cellulose), and lignin have been widely explored for the synthesis or modification of biobased polymers.^3^ Biomass-derived carbon is particularly attractive: Although global biomass stores about 1.2 × 10^11^ tons of carbon, surpassing the 8.0 × 10^10^ tons contained in petroleum, only a small fraction (approximately 5%) is currently exploited.^5^ Within this vast resource, terpenes stand out as plant-derived, isoprene-based (C_5_H_8_) molecules available in large quantities.^4,5^ Limonene has attracted considerable attention in research on renewable materials due to its high natural abundance, particularly in the peels of oranges and other citrus fruits.^4^

Beyond the interest in renewable sources, the sustainability of the polymerization processes themselves has become crucial. While conventional radical polymerization of limonene is hindered by intense chain transfer phenomena,^6^ alternative methodologies have overcome these limitations. Ring-opening polymerization (ROP) and ring-opening copolymerization (ROCOP) of limonene oxides, with CO_2_ or cyclic anhydrides,^7–9^ have enabled the synthesis of high-performance polycarbonates and semiaromatic polyesters with remarkable transparency, high glass transition temperatures, and controlled architectures. Thiol–ene chemistry has further expanded the design of limonene-based thermosets,^4^ recyclable networks,^10^ and shape-memory systems,^11^ while the formation of limonene-derived lactams has opened new avenues toward high-performance polyamides.^12^ These advances illustrate how the chemical versatility of limonene directly supports the development of multifunctional next-generation polymeric materials.

This study provides a comprehensive overview of limonene-based polymerization routes, mapping the evolution from traditional radical systems to finely controlled ionic, coordination, ROCOP/ROP, thiol–ene, and photochemical processes. We highlight not only the resulting polymers, such as poly­(limonene carbonate), semiaromatic polyesters, renewable polyamides, recyclable thermosets, and 3D/4D printing resins, but also the growing potential of limonene as a strategic molecular platform for advanced sustainable materials. By consolidating the mechanistic, synthetic, and application-driven progress of the last decades, this review aims to clarify whether limonene can truly transcend its role as a simple fragrance molecule and establish itself as a cornerstone in the transition toward high-performance, renewable, and circular polymer technologies.

## 2 Limonene Structure, Physicochemical, and Functional Properties

Limonene is a cyclic monoterpene abundantly present in citrus fruit peels and is one of the most common compounds in essential oils of aromatic plants and fruits.^13,14^ This abundance is attributed to its role as a precursor of several monocyclic monoterpenoids.^13^ It is found naturally in two enantiomeric forms, d-limonene (or *R*-(+)-limonene) and l-limonene (or *S*-(−)-limonene), depicted in Figure 1b, which differ in optical activity and exhibit distinct sensory and biological properties.^15,16^ d-Limonene, the most common form, has the characteristic orange odor and is primarily obtained from citrus fruits, whereas l-limonene exhibits a lemon-like fragrance and can also be found in essential oils of pine and mint.^13,17^ Limonene is notable for its unique structure and versatile physicochemical properties, which enable applications across the food, pharmaceutical, and cosmetic sectors.

**Figure 1:**
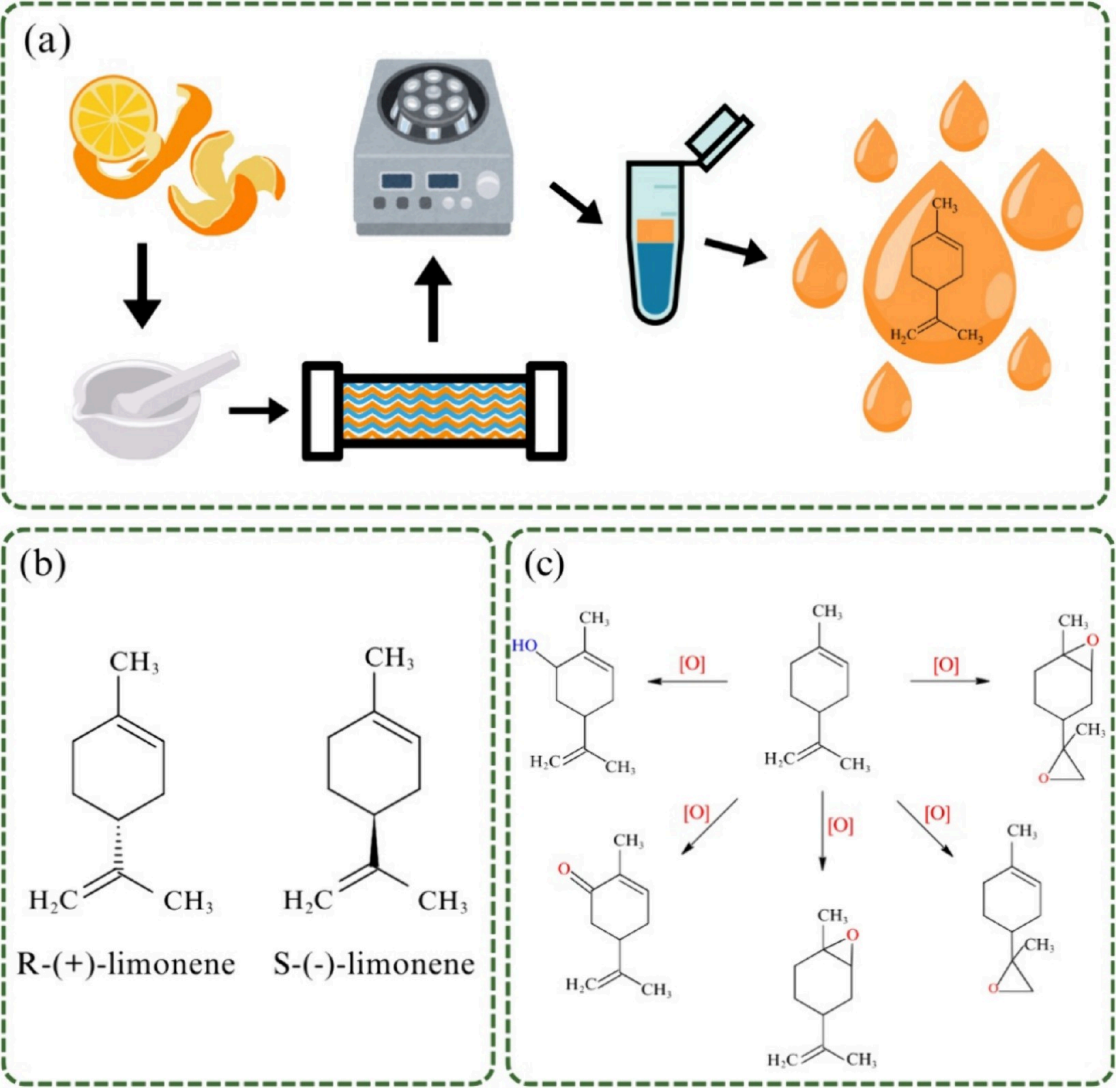
(a) Laboratory-scale isolation of limonene. (b) Limonene enantiomers. (c) Synthesis of limonene-derived oxides, adapted with permission.^17^ Copyright 2021 Elsevier.

To obtain limonene, it is necessary to extract the essential oil from citrus fruits, which may be accomplished by simple processes such as maceration of the peels followed by water-assisted recovery of the released oil, with subsequent separation from water by centrifugation.^18^ From the essential oil, limonene can be separated using techniques such as solvent extraction (e.g., methanol or acetone), supercritical CO_2_ extraction, and microwave or enzyme-assisted methods.^14^ Figure 1a illustrates a simplified laboratory-scale scheme for limonene extraction. Industrially, limonene can be obtained by cold pressing orange peels or by steam-dragging strategies.

The physicochemical properties of limonene, particularly its volatility and lipophilicity, make it highly effective as a flavoring and fragrance agent in the food and cosmetic industries.^19,20^ Its characteristic citrus aroma and high volatility make it an essential component in perfumes and cleaning products, incorporated into a wide range of commercial formulations.^19,21^ Limonene is poorly soluble in water but soluble in organic solvents such as carbon tetrachloride, carbon disulfide, benzene, alcohol, and petroleum ether.^14,17^ Combined with its ability to act as a natural solvent in extraction processes, these features expand its industrial applications.^21,22^ Limonene has a density of 0.8411 g/cm^3^ at 20 °C, a boiling point between 175 and 178 °C, and low toxicity.^14,17^ These properties allow it to substitute toxic organic solvents in some applications, such as replacing *n*-hexane in the extraction of rice bran oil. Another key property of limonene is its optical activity, i.e., its ability to rotate plane-polarized light.^17^

From a structural perspective, limonene contains two double bonds, one exocyclic (outside the ring) and one endocyclic (within the ring). The presence of these unsaturations imparts reactivity toward numerous derivatization reactions. Limonene chirality also plays a crucial role in the synthesis of structurally diverse polymers, influencing the arrangement of functional groups during polymerization and imparting unique functionalities to the resulting materials.^1,23^ A classic example is its oxidation and epoxidation, which enable the synthesis of derivatives such as limonene epoxides,^22,24^ as well as other oxides, as illustrated in Figure 1c. The limonene epoxides in Figure 1c, hereafter referred to as limonene oxides, can serve as monomers in polymerization processes, either through homopolymerization or copolymerization with carbon dioxide or anhydrides, yielding polycarbonates and polyesters with tunable properties.^1,25^

Functionally, limonene exhibits a broad range of biological activities, including antioxidant, anti-inflammatory, and anticancer properties, which make it a promising compound for pharmaceutical applications.^20,26^ Studies indicate that it can stabilize protein structures and inhibit glycation processes, an attractive feature for preventing diseases such as diabetes.^27^ Moreover, its ability to inhibit fungal growth and mycotoxin production highlights its potential as a natural fungicide.^28^ These bioactive properties justify its inclusion in dietary supplements aimed at promoting health and well-being.^20,26^

In parallel, the growing demand for sustainable and renewable resources has stimulated research on microbial production of limonene from agro-industrial residues, reinforcing its role as a compound of interest in the bioeconomy.^29,30^ Metabolic engineering strategies in microorganisms such as *Yarrowia lipolytica* and *Escherichia coli* have been explored to optimize its biosynthesis from renewable feedstocks, demonstrating ongoing efforts to enable industrial-scale production in a more sustainable manner.^19,29,30^ Table 1 provides an overview of the main limonene polymerization routes and a brief description of the resulting products.

**Table 1:** Overview of Limonene Polymerization: from Early Studies to Recent Advances, Highlighting Major Polymerization Routes and the Resulting Products

main reagents	polymerization route	main product	reference
acrylonitrile, limonene, BPO, methanol, and DMSO	radical copolymerization	copolymer with nitrile group	^31^
methyl methacrylate, limonene, BPO, and CHCl_3_	radical copolymerization	poly(limonene-*co*-methyl methacrylate)	^32^
limonene oxide and CO_2_	ring-opening copolymerization	poly(limonene carbonate)	^33^
*N*-vinylpyrrolidone, limonene, AIBN, and methanol	radical copolymerization	copolymer with pyrrolidone group	^34^
limonene, maleimide, AIBN, nonfluorinated alcohol, and DMF	“living” radical copolymerization	poly(limonene-*co*-maleimide)	^35^
limonene, xylene, and BPO	radical polymerization	oligomeric poly(limonene)	^36^
limonene oxides and pinene oxide	cationic ring-opening polymerization	polyether	^37^
BMA, d-limonene, BPO, methanol, CHCl_3_, and THF	radical copolymerization	poly(limonene-*co*-n-BMA)	^38^
methyl 10-undecenoate, TMDS, GaBr_3_, d-limonene, DMPA, anhydrous Na_2_SO_4_, TBD, silica gel, thioacetic acid, H_2_O_2_ solution, and AIBN	thiol–ene copolymerization	polysulfide polyether	^39^
limonene, TMPMP, PETMP, and ethyl acetate	thiol–ene photopolymerization	thermoset coating	^4^
BA, BPO, 1,1-di-(*tert*-butylperoxy)-3,3,5-trimethylcyclohexane (Luperox 231), and limonene	radical copolymerization	poly(limonene-*co*-n-butyl acrylate)	^40^
d- and l-limonene oxide, zinc β-diiminate complex, and *n*-hexane	ring-opening copolymerization	poly(limonene carbonate)	^41^
THF, CH_2_Cl_2_, diethyl ether, activated alumina, LO, polymer mercaptan 358, CO_2_, hexamethylene diisocyanate-based polyisocyanate blocked by caprolactam, and (Et-BDI)Zn[N(SiMe_3_)_2_]	ring-opening polymerization	poly(limonene carbonate)	^42^
triethylamine, *p*-methoxyaniline, chloroacetyl chloride, sodium methacrylate, Tebax, NaI, d-limonene, AIBN, hydroquinone, and 1,4-dioxane	radical copolymerization	poly(limonene-*co*-*p*-methoxyaniline)	^43^
limonene, DMA, benzophenone, thioxanthen-9-one, PMDETA, MBI, EBP, 1BB, and TBE	radical photopolymerization	oligomeric poly(limonene)	^44^
AlClMe_2_, LO, and PLA	catalytic ring opening	polyether	^45^
ethylene, limonene, and pinene	coordination copolymerization	high-molecular-weight poly(ethylene-*co*-limonene) (Mn = 4.09–16.4 × 10^4^ Da)	^46^
styrene, d-limonene, TEMPO, and BPO	nitroxide-mediated radical copolymerization	limonene-based oligomers and functionalized polystyrene, poly(limonene-*co*-styrene)	^47^
6-HDL, PDL, LO, PA, aluminum complex, toluene, CDCl_3_, and PPNCl salt	ring-opening copolymerization	poly[(limonene phthalate)-*block*-poly(pentadecalactone)] and poly[(limonene phthalate)-*block*-poly(hexadecenlactone)]	^48^
β-myrcene and limonene	radical copolymerization followed by thiol–ene photopolymerization	poly(limonene-*co*-myrcene)	^49^
LO, phthalic anhydride (polyester-PHLO), cyclohexene anhydride (polyester-CHELO), cyclohexane anhydride (polyester-CHALO), norbornene anhydride (polyester-NOLO), toluene, DBU, thiourea cocatalyst, and propargylic alcohol initiator	ring-opening copolymerization	polyester	^11^
d-limonene, PETMP, dichloromethane (DCM), and I2959	thiol–ene photopolymerization	recyclable thermoset polymer	^10^
40% methylamine, hexylamine, ethanolamine, 30% ammonium hydroxide solution, limonene, limonene epoxide, benzylamine, aniline, dimethyl carbonate, TBD, DBU, boron trifluoride diethyl etherate, methyl trifluoromethanesulfonate, dichloromethane, diethyl ether, anhydrous sodium sulfate, acetone, CDCl_3_, and DMSO	anionic ring-opening polymerization (AROP)	nonisocyanate polyurethanes	^50^
limonene, ethylene glycol dimethacrylate, calcium chloride hexahydrate, palmitic acid, and potassium persulfate	emulsion radical copolymerization	poly(limonene-*co*-EGDMA) (PLE)	^51^
LO, *trans*-menth-1-ene oxide, and zinc β-diiminate [(BDI)Zn-μOAc]	ring-opening copolymerization	polycarbonate	^52^
limonene diepoxide, acrylic acid, methacrylic acid, *N*-hydroxyethyl acrylamide, potassium persulfate, and AIBN	ring-opening polymerization	poly(meth)acrylates and poly(acrylamide)	^53^
limonene oxide, sodium hydride, Bz(CaLa)_2_, Bz(LiLa)_2_, Imes, and P_4_-*t*-Bu	anionic ring-opening polymerization (AROP)	limonene polyamide	^54^

Table 1 broadly demonstrates the versatility of limonene polymerization, with recent emphasis on copolymerization routes involving oxide or thiol–ene reactions. These processes can be conducted through various routes and methodologies, yielding products with diverse properties, ranging from smaller structures such as oligomeric poly­(limonene) to more complex systems such as branched or cross-linked copolymers. The following sections discuss different polymerization routes and methodologies for limonene, as well as the main products and applications, with particular emphasis on those most promising in the current context of renewable polymer synthesis.

## 3 Addition Polymerization and Copolymerization of Limonene

### 3.1 Radical Polymerization

The most traditional route for synthesizing polymers from unsaturated monomers is radical addition polymerization. However, one of the main challenges in the radical polymerization of limonene is its strong tendency toward chain transfer reactions, which can significantly affect both the molecular weight and the properties of the resulting polymer. The presence of allylic C–H bonds in limonene makes it particularly susceptible to such reactions, as hydrogen abstraction at the allylic position readily generates resonance-stabilized radicals that favor chain transfer over propagation, leading to premature chain termination and products with low molecular weight.^38,55^ This mechanistic feature intrinsically limits molecular-weight build-up and typically results in broad dispersities, even under conditions optimized for conventional free-radical polymerization.^56–58^ For instance, Singh and Kamal (2012) demonstrated that the radical polymerization of d-limonene using benzoyl peroxide (BPO) as initiator resulted in nonideal kinetic behavior, with the polymerization rate influenced by both primary radical termination and degradative chain transfer.^36^ This behavior was further supported by Zhang and Dubé (2014), who observed that the chain transfer mechanism competes with chain propagation, ultimately yielding lower molecular weights than expected.^38^ Together, these studies establish allylic hydrogen-induced chain transfer as a fundamental mechanistic limitation of limonene radical polymerization rather than a purely process-dependent effect.

Some studies have reported the copolymerization of limonene with acrylates. For example, Ren et al. (2015) investigated the copolymerization of limonene with *n*-butyl acrylate (BA) via free-radical polymerization using BPO as the initiator. They found that BA was more reactive than limonene and that the glass transition temperature (*T*_g_) of the resulting copolymer increased with the incorporation of limonene, which was considered advantageous for applications. However, increasing the limonene fraction led to lower molecular weight and reduced dispersity, attributed to chain transfer caused by the allylic hydrogens in limonene.^40^ This trade-off between renewable monomer incorporation and molecular-weight control highlights the need for strategies that mitigate allylic chain transfer while preserving the limonene content. In a related study, Ren et al. (2015) examined the free-radical terpolymerization of BA, butyl methacrylate (BMA), and limonene and were able to predict the final composition using copolymerization models. They observed that limonene did not homopolymerize but was incorporated into the terpolymer only in the presence of BA and BMA.^55^

Sharma and Srivastava (2003, 2004) analyzed the polymerization of limonene with methyl methacrylate (MMA) in separate studies. In one of them, they investigated the reaction mechanism and kinetics of limonene and MMA polymerization using BPO as the initiator, showing a strong tendency toward alternating copolymerization. In this case, limonene was responsible for polymer functionalization, but the higher polymerization rate was only achieved with increased MMA content.^32^ In another study, they examined the free-radical terpolymerization of limonene, styrene, and MMA, also initiated with BPO. They found that MMA was more reactive than both limonene and styrene.^59^ These results further reinforce that limonene predominantly acts as a comonomer or functional modifier in radical systems rather than as an efficient chain-propagating monomer.

Targeting the mitigation of allylic chain transfer phenomena that limit molecular weight development in limonene radical polymerizations, De Oliveira and Vieira (2020) employed halogenated initiators and photoinitiators in the controlled radical photopolymerization of limonene. The resulting polymer exhibited gum-like characteristics with low dispersity, and the highest molecular weight was achieved using 2,2,2-tribromoethanol (TBE) as initiator, which also improved conversion with increasing concentration.^44^ These findings suggest that initiator structure and radical generation pathways can partially mitigate, but not fully suppress, allylic chain transfer effects, as confirmed in another study from the same research group.^60^ Additionally, the nitroxide-mediated polymerization (NMP) of styrene and limonene was studied by Andriotis et al. (2021), who found poor copolymerization efficiency with low limonene incorporation into the resulting copolymer and formation of some oligomers. In this case, limonene acted as a radical “inhibitor”, suppressing the thermal autopolymerization of styrene. Nevertheless, the authors emphasized that limonene-functionalized polystyrene holds promise for new applications while introducing a renewable fraction into the macromolecular structure.^47^

Kekevi et al. (2024) synthesized a limonene-based polymer with ethylene glycol dimethacrylate (EGDMA) using high internal phase emulsions (HIPEs), obtaining poly­(limonene-*co*-EGDMA) (PLE) (Figure 2a). The polymer was purified in two ways: Soxhlet extraction with ethanol yielded PLE-E, and lyophilization yielded PLE-L. These polymer matrices were impregnated with palmitic acid (PA), producing PA@PLE-E and PA@PLE-L composites. As shown in Figure 2b, the composites exhibited ring-shaped porous structures. The authors reported differences in permeability depending on the purification method, with PLE-E being more permeable than PLE-L. Differential scanning calorimetry (DSC) analysis indicated that these composites are promising for thermal energy storage applications, particularly in passive solar heating systems.^51^

**Figure 2:**
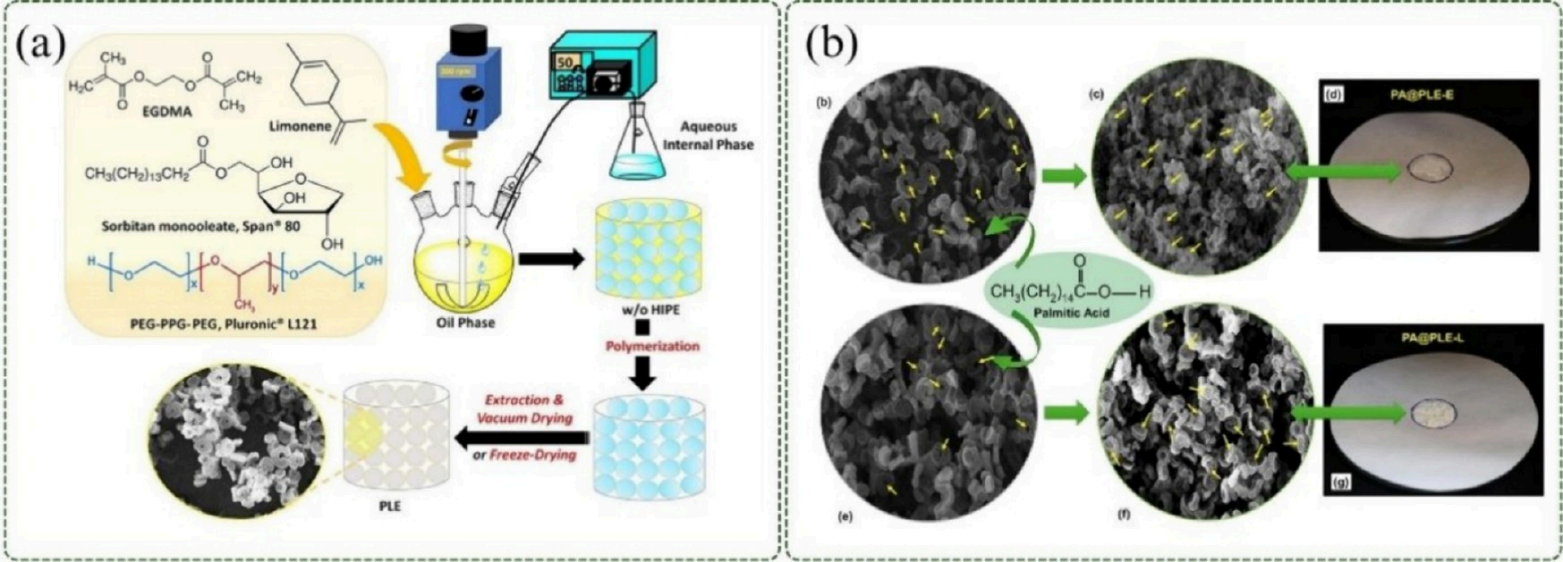
(a) Schematic representation of limonene polymerization with EGDMA and formation of the PA-based composite, adapted with permission.^51^ Copyright 2024 Elsevier. (b) Optical microscopy images of PLE and the PLE–palmitic acid composite, adapted with permission.^51^ Copyright 2024 Elsevier.

Although promising from the perspective of renewable monomer utilization, radical polymerization of limonene faces persistent challenges related to chain transfer reactions, which limit control over the molecular weight and dispersity of the resulting polymers. These intrinsic mechanistic constraints have motivated the exploration of alternative polymerization strategies as well as monomer and process modifications, aimed at decoupling limonene incorporation from allylic chain transfer pathways. In this context, alternative addition polymerization routes, such as ionic (cationic and anionic) mechanisms, emerge as promising approaches to overcoming these constraints.

### 3.2 Ionic Polymerization

Ionic polymerization is a synthesis method that enables precise control over the polymer structure, molecular weight, and functional properties. This technique can be broadly categorized into two types: cationic and anionic polymerization. Each of these approaches employs ionic species as active centers for chain growth, resulting in high selectivity and polymers with well-defined characteristics. Cationic polymerization involves the use of reactive cations, such as those derived from Lewis acids, or proceeds through electrophilic mechanism.^61^ In contrast, anionic polymerization relies on the stabilization of propagating anionic species, which can be strongly influenced by solvent choice.^62^ Ionic routes have already been explored for the synthesis of various terpene-derived polymers,^62–66^ although their application to the polymerization of limonene itself remains largely underexplored. In such cases, limonene is typically employed as a (co)­monomer.

For instance, the copolymerization of limonene with lacol demonstrated the potential for the development of sustainable materials with enhanced mechanical properties and high radiation resistance in the study by Arachchilage et al. (2020). Specifically, cationic polymerization was employed using AlCl_3_·EtOAc as a (co)­initiator, yielding copolymers with different limonene contents. The copolymers exhibited *T*_g_ modulated by the limonene fraction, indicating improved thermal stability compared with pure lacol. These authors further subjected the copolymers to γ radiation, which induced cross-linking, confirmed by the emergence of new —C—O—C— linkages and increased hardness, without evidence of degradation. These findings highlight limonene not only as a sustainable component for the synthesis of homopolymers but also as a reinforcing agent that enhances mechanical strength and radiation resistance, rendering such copolymers promising for advanced applications such as radiation shielding and durable coatings.^67^

### 3.3 Coordination Polymerization or Copolymerization

Coordination polymerization is a classical route that involves chain growth through the coordination of monomers to a metal catalyst, typically a transition metal, followed by insertion into a metal–carbon bond. This mechanism is fundamental to the production of polyolefins, such as polyethylene and polypropylene, enabling precise control over molecular weight, tacticity, and branching. Catalysts such as Ziegler–Natta, metallocenes, and postmetallocenes are commonly employed, each offering distinct capabilities for stereoregularity and material properties. This method is widely used in industrial applications due to its efficiency and ability to yield polymers with tailored mechanical properties and high crystallinity.^68^

The first report of stereoregular terpene polymerization (myrcene) dates back to 1960, employing heterogeneous Ziegler–Natta catalysts to obtain higher molecular weight polymers at 0 °C with monomer conversion around 80%.^69^ During the same decade, ethylene–propylene terpolymers (EPT) incorporating limonene and β-pinene as third monomers were also explored by using various catalysts of this type. The goal in this case was to produce elastomers with improved properties through the introduction of these monoterpenes. The coordination polymerization was conducted by vapor-phase introduction of terpene monomers into the ethylene–propylene gas stream, with catalytic systems including triethylaluminum (AlEt_3_), triisobutylaluminum (Al­(i-Bu)_3_), and diisobutylaluminum chloride (Al­(i-Bu)_2_Cl), combined with transition metal compounds such as vanadium oxytrichloride (VOCl_3_), titanium tetrachloride (TiCl_4_), titanium tetraiodide (TiI_4_), and vanadium triacetylacetonate (V­(acac)_3_). The molecular weights of the resulting terpolymers, determined via intrinsic viscosity measurements in benzene, ranged from 4.14 × 10^5^ to 1.53 × 10^6^ Da (g/mol) depending on the catalytic system and reaction conditions. Limonene incorporation into the polymer matrix varied widely, reaching up to 20.5% in the most efficient reactions.^70^

More recently, the copolymerization of ethylene with limonene and β-pinene, mediated by modified titanocene catalysts in combination with methylaluminoxane, enabled the synthesis of high-molecular-weight poly­(ethylene-*co*-limonene) and poly­(ethylene-*co*-pinene) copolymers. The poly­(ethylene-*co*-limonene) copolymers exhibited molecular weights ranging from 4.09 to 16.4 × 10^4^ Da (g/mol). Catalytic activity and copolymer properties were modulated by the Al/Ti ratio and polymerization temperature, although these parameters showed little effect on comonomer incorporation or final molecular weight. This study emphasized the critical role of catalyst design in controlling the microstructure and final properties of copolymers, opening new opportunities for the development of semicrystalline polyolefins with specific functionalities.^46^

Based on these findings, the use of stereospecific catalysts in the coordination polymerization of limonene can be considered effective for the synthesis of copolymers with significantly high molecular weights. Nevertheless, its application remains largely underexplored, as chemically modified limonene derivatives, combined with ionic and coordination polymerization routes, have emerged as strategic approaches to overcome the limitations observed in conventional addition polymerizations. This combination of strategies may enable the synthesis of high-value materials with tunable properties and greater sustainability. For instance, as highlighted in this review, limonene can be readily epoxidized (Figure 1c), generating highly reactive groups that substantially expand the range of synthetic routes and applications in the field of sustainable materials.

## 4 Limonene Epoxidation and Ring Opening

### 4.1 Epoxidation Reaction

The epoxidation of limonene is a chemical reaction that converts the double bonds present in the limonene molecule into epoxide (or oxirane) groups through the addition of an oxygen atom to the unsaturated system. As previously discussed, limonene is a monoterpene containing two double bonds, one exocyclic and one endocyclic (Figure 1b). Epoxidation generally occurs at these double bonds, forming monoepoxides or diepoxides, depending on the reaction conditions and the oxidizing agent employed. This reaction can be categorized into chemical, biological, or enzymatic methods.^71^ Regarding chemical methods, different oxidizing agents can be used, such as hydrogen peroxide combined with solvents including acetone, ethyl acetate, ethanol, or chloroform. This method may yield various products depending on the solvent type and concentration, catalyst properties, and reaction temperature.^72^ Figure 3 illustrates representative products obtained from the epoxidation of limonene using hydrogen peroxide as the oxidizing agent.

**Figure 3:**
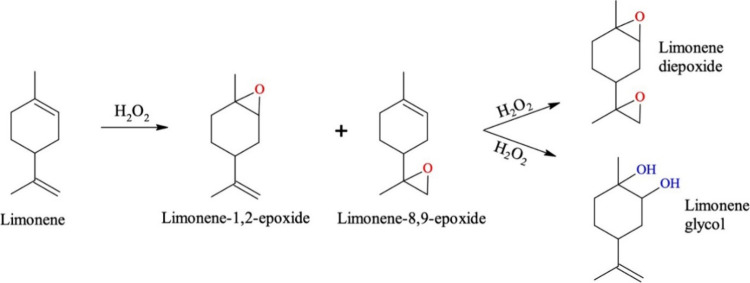
Limonene epoxidation with hydrogen peroxide, adapted with permission.^76^ Copyright 2023 American Chemical Society.

A widely used approach involves titanium-based catalysts, such as Ti-MCM-41 and Ti-SBA-15, combined with hydrogen peroxide (H_2_O_2_) as the oxidant. These catalysts exhibit high activity and selectivity toward the formation of limonene-1,2-epoxide. For instance, the epoxidation can be conducted at approximately 70 °C with an optimized molar ratio between limonene and hydrogen peroxide, ensuring maximum yield.^73^ Another effective strategy relies on biocatalysts, such as immobilized lipase *Candida antarctica* *lipase B* (CALB), which has shown high yields of limonene epoxide under mild conditions. In a recent study, the reaction was performed at 50 °C with 40 mM limonene and 250 mM H_2_O_2_, achieving around 75% of the desired product in only 40 min.^74^ This biocatalytic approach stands out as environmentally friendly and offers significant potential for process optimization. Furthermore, chemoselective epoxidation can be achieved using organometallic catalysts, such as manganese (Mn) complexes, which promote the selective formation of limonene epoxides with high regioselectivity.^75^

Another strategy involves the synthesis of polycarbonates via limonene epoxidation using modified Y zeolites as heterogeneous catalysts, as reported by Gallego-Villada et al. (2024). The most effective catalyst was KSnHYD2, a potassium- and tin-modified Y zeolite, which provided 97% conversion at 70 °C with high selectivity toward monoepoxides.^72^ Michel et al. (2012) investigated the epoxidation of limonene using methyltrioxorhenium (MTO) as a catalyst, targeting the selective production of limonene-1,2-oxide. Optimal reaction conditions were achieved with dichloromethane (CH_2_Cl_2_) at 25 °C, employing 0.5 mol % MTO, yielding 96% selectivity to the desired product.^77^ Another catalytic method was reported by Egusquiza et al. (2012), employing heteropolytungstates containing copper­(II) and with hydrogen peroxide as the oxidant. The PWCu catalyst exhibited the best performance, achieving 96.1% conversion and 89% selectivity toward the epoxide after 40 h.^78^ Moreover, the authors emphasize the replacement of chlorinated solvents with acetonitrile as a more sustainable alternative.

Limonene epoxidation can also be conducted in acetone or other organic solvents, and the solvent choice strongly influences the kinetics and selectivity of the reaction. According to Gallego-Villada et al. (2023), the best-performing solvents were ethyl acetate, ethanol, and toluene, depending on the catalytic system employed. In the same study, the authors summarized catalytic systems and their corresponding conversions.^76^ Table 2 provides an overview of the catalytic systems reported by these authors that achieved conversions above 90% and selectivity for epoxide or diepoxide formation above 60%.

**Table 2:** Catalytic Oxidation Systems of Limonene, Achieving Conversions Higher than 90%, and Selectivities toward Epoxides or Diepoxides Exceeding 60%, Adapted from Gallego-Villada et al. (2023)^72^

catalyst	temperature (°C)	time (h)	oxidizing agent	solvent
aluminum oxide (Al_2_O_3_)	80	10	H_2_O_2_	ethyl acetate
Mo-TUD-1	70	24	*tert*-butyl hydroperoxide (TBHP)	α,α,α-trifluorotoluene (TFT)
MnFe_2_O_4_/Phttpy-MoO_2_	95	0.5	*tert*-butyl hydroperoxide (TBHP)	solvent-free
[MoO_3_(biim)]	70	6	*tert*-butyl hydroperoxide (TBHP)	α,α,α-trifluorotoluene (TFT)
ZnAl-LDH-BIAN-MoI_2_	110	24	*tert*-butyl hydroperoxide (TBHP)	toluene

Limonene epoxides represent versatile intermediates for the synthesis of new materials due to the high reactivity of the oxirane ring. These compounds can undergo ring-opening reactions with various nucleophiles, enabling the introduction of diverse functional groups and the construction of polymeric structures with tunable properties. This approach allows for the development of materials with potential applications in packaging, coatings, and biomaterials. The following sections present different classes of polymers derived from limonene epoxides, highlighting their synthetic routes, physicochemical properties, and possible applications.

### 4.2 Synthesis of Polycarbonates

The synthesis of polycarbonates from limonene represents a biobased, sustainable, and promising route for producing high-performance plastics with excellent thermal and optical properties. These polycarbonates are more transparent and harder than those traditionally produced from bisphenol A (BPA), a carcinogenic compound that has raised numerous concerns in the synthesis of commercial polycarbonates.^52,79^ The most compelling aspect of this route lies in the copolymerization of limonene oxide (LO) with carbon dioxide (CO_2_), as schematically illustrated in Figure 4a, which contributes to carbon capture and storage through two complementary pathways: the use of a renewable monomer combined with CO_2_. This route generally requires specific catalysts and high pressures.

**Figure 4:**
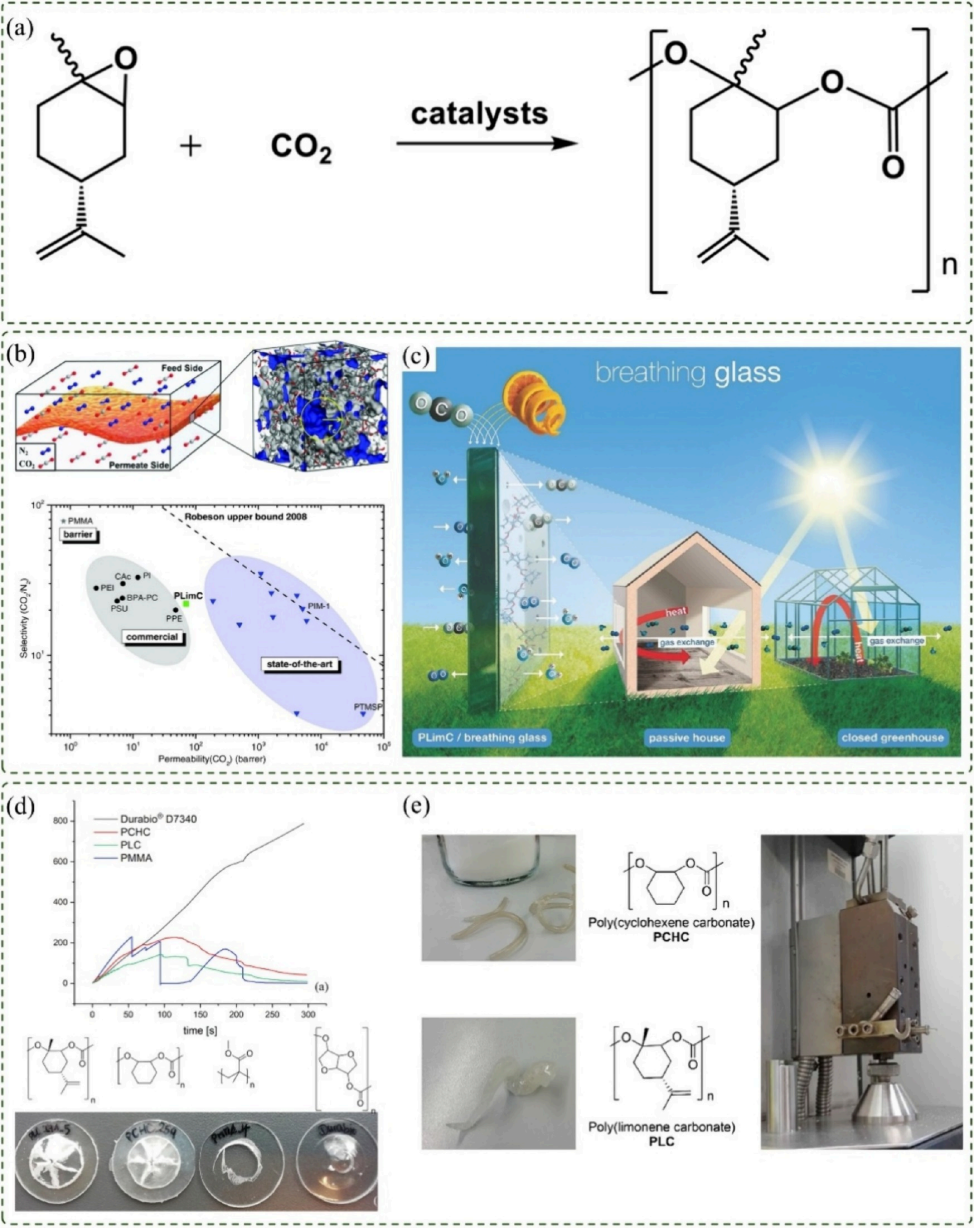
(a) General scheme of limonene polycarbonate (PLimC) synthesis, adapted with permission.^79^ Copyright 2021 Elsevier. (b) Robeson plot for the CO_2_/N_2_ gas pair with PLimC compared to commercial and state-of-the-art polymeric membrane materials, including cellulose acetate, polyimide, poly­(ether imide), polysulfone, and poly­(phenylene ether), adapted with permission.^81^ Copyright 2017 John Wiley and Sons. (c) Schematic representation of a highly permeable glazing material, applicable in passive house windows to reduce energy consumption or in greenhouses to minimize water loss through continuous ventilation,^81^ adapted with permission. Copyright 2017 John Wiley and Sons. (d) Multiaxial tensile test of cylindrical specimens showing stress (MPa) vs time (s) along with fracture patterns of the tested polymers, adapted with permission.^82^ Copyright 2020 Elsevier. (e) Images of extruded PCHC at 180 °C and PLimC at 175 °C, adapted with permission.^82^ Copyright 2020 Elsevier.

Hauenstein et al. (2016) reported one of the first examples of high-molecular-weight poly­(limonene carbonate) (PLimC), exceeding 100 × 10^3^ Da, with excellent transparency (94% visible-light transmission) and low turbidity, via copolymerization of LO and CO_2_ using a zinc β-diiminate catalyst. The resulting polymer exhibited a *T*_g_ of 130 °C, close to that of commercial BPA-based polycarbonate. Control of the molecular weight was achieved by optimizing monomer–catalyst ratios and removing hydroxyl-containing impurities via iodomethane treatment, enabling the production of PLimC with number-average molar masses up to 109 × 10^3^ Da and narrow dispersity (1.10–1.19). Batch-scale production exceeding 1 kg demonstrated the scalability of the process. Kinetic studies further revealed second-order dependence on LO, zero-order on CO_2_, and first-order on the catalyst, supporting a mechanism involving alternating LO and CO_2_ insertion mediated by the catalytic system, thus producing an alternating copolymer.^80^

In terms of barrier properties, a complementary study by Hauenstein et al. (2017) demonstrated that PLimC exhibits high CO_2_ permeability and favorable selectivity relative to N_2_ (CO_2_/N_2_ ≈ 19), comparable to commercial membranes (Figure 4b). Heating the material to 80 °C further increased the CO_2_ permeability, making it attractive for gas separation processes. Moreover, PLimC showed promising performance in thermal insulation and mechanical strength tests. This unique combination of optical properties, gas permeability, and structural robustness inspired the authors to propose its use as “breathable glass”. Unlike conventional glass, which is impermeable to gases, PLimC would enable passive gas exchange while maintaining transparency and thermal insulation, making it attractive for greenhouses or residential applications (Figure 4c). In such environments, the material could reduce or even eliminate the need for energy-intensive ventilation systems by enabling the diffusion of CO_2_ through glazing. Compared with conventional membrane polymers, PLimC stands out for its transparency, thermoplasticity, and sustainability, positioning it as a promising candidate for both CO_2_-capture membranes and breathable transparent materials for sustainable architecture.^81^

Kernbichl and Rieger (2020) synthesized and compared poly­(cyclohexene carbonate) (PCHC) and PLimC with other traditional polymers, demonstrating an attractive balance of thermal stability, mechanical resilience, and processability. Both polymers withstand temperatures up to 150 °C with minimal decomposition, although PLimC exhibits a tendency toward cross-linking at higher temperatures, which can be mitigated by hydrogenation or antioxidant additives. Mechanically, PCHC and PLimC offered intermediate performance between brittle poly­(methyl methacrylate) (PMMA) and highly impact-resistant Durabio (Figure 4d). Unlike PMMA, the tested polycarbonates did not shatter upon fracture, indicating a more ductile behavior. Under multiaxial pressure tests, both materials distributed force evenly, with PCHC displaying higher strength and PLimC greater flexibility. Furthermore, PCHC could be processed at 180 °C with minimal degradation when stabilized, whereas PLimC required lower processing temperatures (165 °C) (Figure 4e).^82^

Recently, different approaches have been explored to functionalize PLimC, thereby expanding its application potential. For instance, the introduction of peptide chains via ring-opening polymerization of lysine *N*-carboxyanhydride was achieved by incorporating primary amine groups through thiol–ene “click” chemistry, resulting in graft copolymers with varying oligopeptide densities. This strategy yielded highly hydrophilic hybrid materials with water solubility and pH-responsive behavior, properties absent in native PLimC. The authors suggested that such features make these materials attractive for applications in controlled release systems, bioactive coatings, nanocarriers, and stimuli-responsive materials. This transformation converted PLimC, originally apolar and water-insoluble, into a versatile platform for developing bioinspired materials with high renewable content.^83^ Thus, this study not only introduced a novel synthetic pathway but also significantly broadened the scope of PLimC applications in biotechnology, regenerative medicine, and sustainable high-performance materials.

Neumann et al. (2021) investigated the copolymerization of LO with lactide (LA) using the catalyst [(BDI)­Zn-(μ-OAc)], aiming to improve PLimC properties. The one-pot LO/CO_2_ and LA systems yielded block copolymers (PLimC-*b*-PLA), rather than random copolymers. Variation of the LO/LA feed ratio directly affected the final composition, with LA incorporation exceeding the initial feed ratio. To obtain well-defined block copolymers, a sequential ring-opening living polymerization strategy was employed, producing a block of PLimC first, followed by LA insertion in order to obtain a second block. The resulting copolymers exhibited high molar mass and characteristic phase-separated behavior with two distinct glass transitions (*T*_g_ of PLimC = 121 °C and *T*_g_ of PLLA = 56 °C). Incorporating lactides functionalized with long alkyl chains produced polyester segments with a reduced *T*_g_ of −38 °C, imparting elastomeric behavior.^9^ These results demonstrate a promising route for fine-tuning PLimC properties via block copolymerization, thereby broadening its potential as a renewable, sustainable material.

A similar copolymerization route was reported by Höferth et al. (2024) to produce a terpolymer with a defined number of functional groups along its backbone. As in the work of Hauenstein et al. (2016), LO, CO_2_, and the zinc β-diiminate catalyst were employed, but with the addition of *trans*-menth-1-ene oxide (Men1O), a compound structurally similar to LO and also derivable from limonene. The difficulty in controlling PLimC functionalization without Men1O justified this strategy. The resulting poly­(limonene carbonate-*ran*-menthene carbonate) [P­(LimC-*ran*-Men1C)] exhibited *T*_g_ values between 130 and 133 °C, comparable to those of PLimC, while providing pendant double bonds for further functionalization. Thiol–ene reactions with 2-mercaptoethanol and 2,2′-azobis­(2-methylpropionitrile) (AIBN) as the thermal initiator yielded P­(LimC-*ran*-Men1C–OH), containing hydroxyl groups distributed in a controlled manner along the polymer chain. These groups enable subsequent polymerization by reversible deactivation radical processes or ring-opening of lactones and lactides, affording new copolymer structures.^52^

### 4.3 Synthesis of Polyesters and Polyethers

Limonene oxide (LO) has demonstrated promising reactivity in cationic ring-opening photopolymerizations, although it is less reactive than α-pinene oxide. Unlike the latter, which undergoes simultaneous opening of both the epoxide and cyclobutane rings, LO polymerizes primarily through its epoxide group. Despite its lower reactivity, LO photopolymerization produced more viscous polymers with higher yields and molecular weights compared with α-pinene oxide. Gel permeation chromatography (GPC) analysis revealed the formation of polydisperse low-molecular-weight oligomers (270–1700 Da) with a bimodal distribution, with polymer yields ranging from 68 to 75% when Rhodorsil 2074 was used as a photoinitiator. Furthermore, the reaction temperature played a critical role in determining the balance between polymerization and isomerization, with lower temperatures favoring ring-opening polymerization and higher temperatures promoting side reactions.^37^

Complementarily, Sessini et al. (2020) investigated the catalytic ring-opening polymerization of LO using an aluminum derivative supported by bulky phenolic ligands, yielding low-molecular-weight poly­(limonene ether) (PLO) with stable thermal properties (Figure 5a). Although the reaction preferentially involved the epoxide of the *cis*-isomer, its efficiency was limited by side-product formation, resulting in oligomers with molar masses up to 1300 Da and moderate dispersities (1.37–1.42). Nevertheless, incorporation of only 10 wt % PLO into PLA films improved matrix flexibility, thermal stability, and hydrophobicity, highlighting the potential of PLO as a renewable-resource-derived plasticizer (as evidenced by *T*_g_ reduction in DSC (Figure 5a). Miscibility and morphological studies confirmed good compatibility at moderate PLO contents, while higher loadings led to phase separation. These findings underscore the relevance of LO as a versatile platform for sustainable polymer additives, despite challenges related to molar mass control and side reactions.^45^

**Figure 5:**
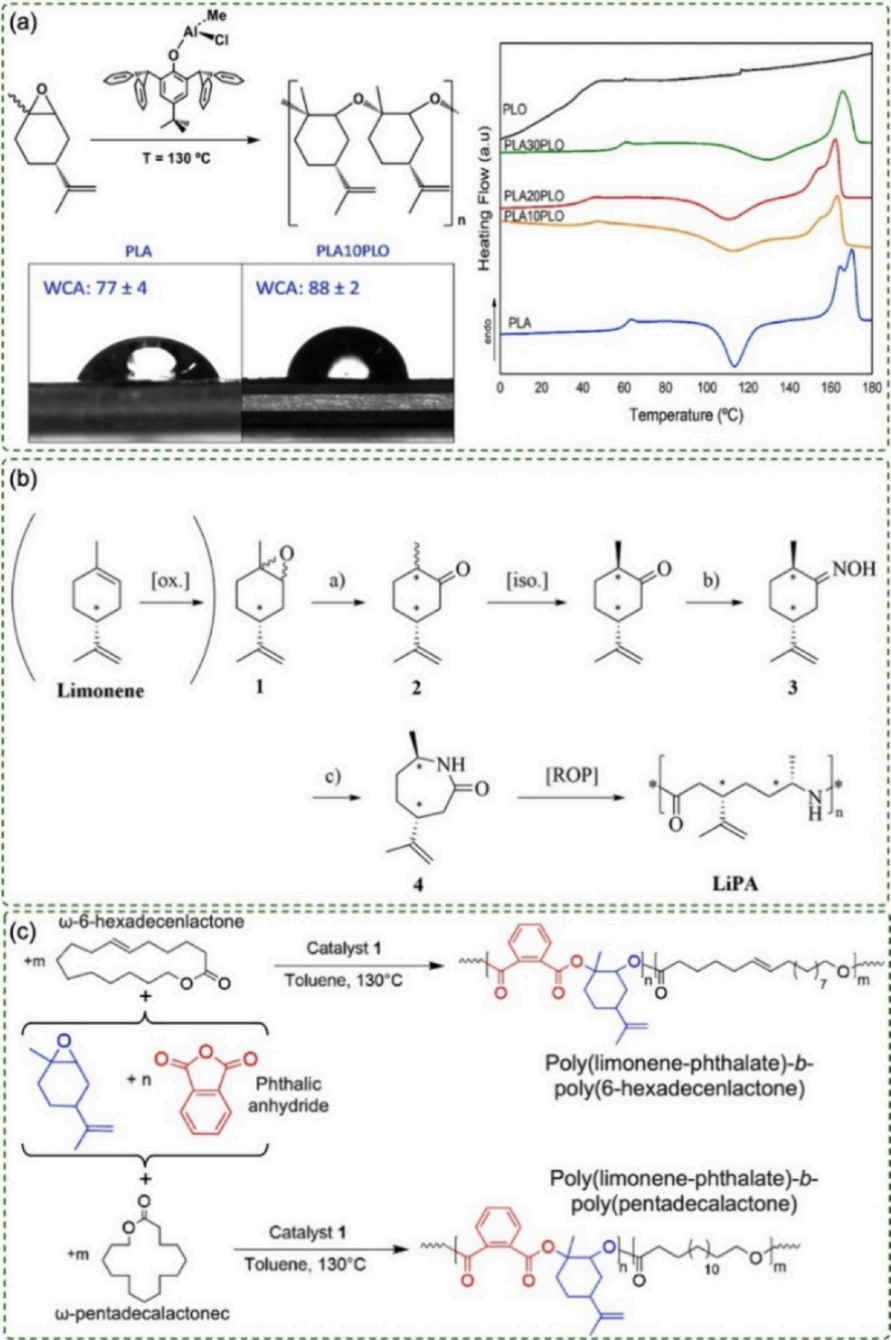
(a) Synthesis of polyethers, polyamides, and polyesters derived from limonene via ROP and ROCOP, and modification of PLA properties through the use of poly­(limonene ether) (PLO) as a plasticizer, adapted with permission.^45^ Copyright 2020 Elsevier. (b) Production of renewable polyamides from structural modifications of limonene.^12^ (c) Synthesis of semi*-*aromatic diblock copolyesters with linear segments obtained from renewable lactones, reproduced from ref 48. Available under a CC-BY 4.0 license. Copyright 2022 D’Auria et al.

Additionally, Nejad et al. (2013) investigated the ring-opening copolymerization of LO with phthalic anhydride (PA) using salophen-type metal complexes, with the Cr–salophen/PPN^+^Cl^–^ system showing the highest catalytic activity. Under bulk conditions (130 °C, 3 h), LO conversions up to 94% were achieved, yielding polyesters with molecular weights in the 5 × 10^3^–9.7 × 10^3^ Da (g mol^–1^) range and low dispersity (PDI ≈ 1.4). MALDI-TOF-MS analysis confirmed the formation of well-defined alternating microstructures, typical of mechanisms governed by rapid chain transfer events. The use of different chain transfer agents (alcohols, acids, and diamines) enabled modulation of the final molar mass without significant loss of activity, while *T*_g_ values varied between 29 and 82 °C, reflecting the influence of composition and chain length.^7^

D’Auria et al. (2022) introduced a one-pot terpolymerization strategy involving LO, PA, and macrolactones, catalyzed by bimetallic aluminum complexes (Figure 5c). This system promoted the formation of semi-aromatic diblock polyesters composed of ring-opening copolymerization (ROCOP) blocks of LO/PA linked to polyethylene-type segments derived from the ring-opening polymerization (ROP) of macrolactones, achieving molar masses up to 6.5 × 10^3^ Da (6.5 kDa) with monomodal distributions (PDI < 1.3). DOSY-NMR confirmed the block architecture, while thermal analysis revealed tunable glass transition temperatures ranging from −54 to 80 °C, depending on the macrolactone incorporation ratio. These results broaden the synthetic versatility of LO as a platform for complex polyesters, overcoming homopolymerization limitations and enabling materials with tailored properties for packaging and engineering applications.^48^

### 4.4 Synthesis of Polyamides

In the field of renewable-resource-derived polyamides, Firdaus and Meier (2013) demonstrated the feasibility of limonene functionalization through thiol–ene addition of cysteamine, yielding renewable diamines capable of acting as AA-type monomers for polyamide synthesis and as precursors for isocyanate-free polyurethanes. These monomers were copolymerized with various renewable diesters, resulting in partially terpenic polyesteramides and polyamides with molar masses up to 12 × 10^3^ Da and thermal properties modulated by the type of comonomer employed. The strategy involved solvent-free routes, organocatalysis, and vacuum conditions, aligning with the principles of green chemistry and highlighting the potential of limonene as a versatile molecular platform for bioinspired polymeric chains.^84^

Complementarily, Kleybolte et al. (2022) reported an efficient route for the synthesis of limonene-lactam, enabling the production of high-performance polyamides (LiPA) through ring-opening polymerization while preserving the isopropenyl group for further chemical modifications (Figure 5b). The resulting polymers achieved molar masses up to 22 × 10^3^ Da, displaying thermal stability above 400 °C and *T*_g_ values around 100 °C, with no detectable melting point due to the steric bulk of the pendant groups. This sustainable synthetic route reinforces the viability of limonene as a renewable feedstock for high-performance technical polyamides.^12^

More recently, Kleybolte et al. (2025) advanced this approach by promoting the thiol–ene functionalization of LiPA side chains, which will be discussed further throughout this text, allowing the introduction of functional groups such as alkyl, ester, and sulfonate moieties. This modification enabled the tuning of crucial properties, including solubility, hydrophobicity, glass transition temperature (44–133 °C), and amphiphilic behavior, with particular emphasis on the formation of stable micelles in aqueous solution from sulfonated LiPA, indicating potential applications in drug-delivery systems. Furthermore, the high tolerance of the thiol–ene reaction to diverse functionalities opens avenues for future incorporation of bioactive peptides, expanding the biomedical application scope.^54^ It is also worth noting that the advances reported by Kleybolte et al. (2024) in the development of high-molecular-weight polyamides from β-pinene lactams, employing anionic ring-opening polymerization (AROP) with different initiators, conceptually resemble the strategy discussed here for limonene-based polyamides, particularly regarding the preservation of unsaturated groups for subsequent functionalization and the structural control aimed at high-performance applications.^85^

## 5 Functionalization of Limonene with Acrylate or Methacrylate Moieties for Partially Biobased Poly(meth)acrylate Synthesis

von Czapiewski et al. (2016) employed a catalytic route to synthesize poly­(acrylates) from structurally modified limonene. Initially, the exocyclic double bond was functionalized through an acetoxylation reaction using palladium­(II) acetate as the catalyst, para-benzoquinone as the oxidant, and a dimethyl sulfoxide (DMSO)/acetic acid solution. The resulting acetoxylated product was saponified to generate an allylic alcohol, which was subsequently converted to an aldehyde using methanol and 1,5,7-triazabicyclo[4.4.0]­dec-5-ene (TBD), followed by catalytic isomerization with palladium hydroxide on carbon. To obtain acrylate monomers, the aldehyde was reacted with acrylic acid and various isocyanates, which were then polymerized using AIBN as an initiator, yielding poly­(acrylates). The resulting polymers exhibited molar masses ranging from 12.8 to 29.8 × 10^3^ Da and glass transition temperatures between 86 and 143 °C, depending on the isocyanate employed. The authors further emphasized the potential applicability of this methodology to other terpenes, thereby broadening the diversity of accessible monomers and obtained polymers.^86^

Araruna et al. (2024) investigated the ring-opening reaction of limonene diepoxide (LD) with acrylic acid (AA), methacrylic acid (MA), and *N*-hydroxyethyl acrylamide (HEAA) to obtain monomers for subsequent polymerization, as illustrated in Figure 6a. The polymerization was carried out in mini-emulsions using different stabilizers and two thermal initiators, potassium persulfate (KPS) and AIBN. These distinct conditions enabled the synthesis of two poly­(meth)­acrylates and one poly­(acrylamide) hydrogel, which exhibited variable porosity, good thermal stability, and biodegradability in the presence of *Comamonas* sp.^53^

**Figure 6:**
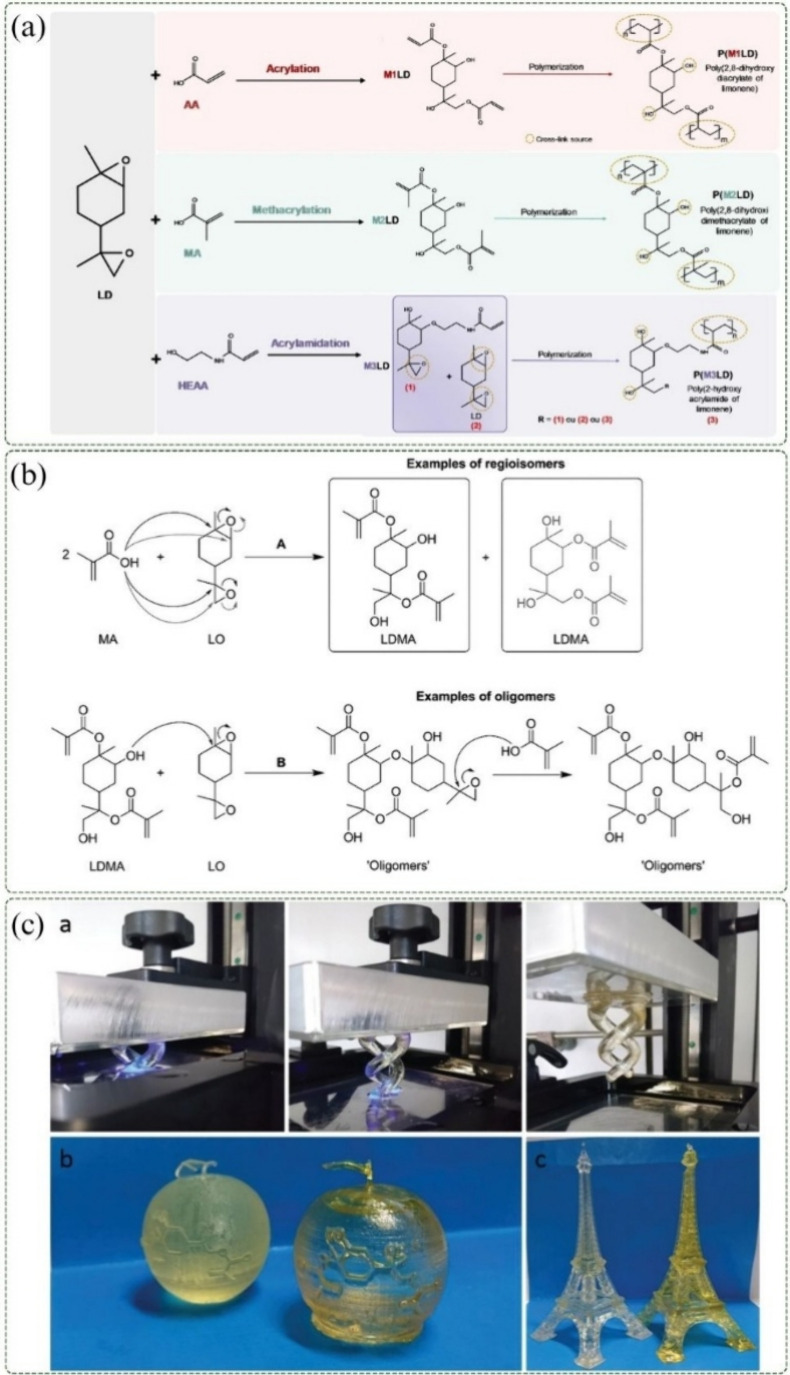
(a) Formation of limonene-based monomers with acrylate (M1LD), methacrylate (M2LD), and acrylamide (M3LD), followed by polymerization, adapted with permission.^53^ Copyright 2024 Springer Nature. (b) Reaction of limonene dioxide with methacrylic acid and formation of possible LDMA oligomers. (c) 3D printing using BisGMA and LDMA, reproduced form ref 87. Available under a CC-BY 4.0 license. Copyright 2020 Schimpf et al.

To replace bisphenol A (BPA)-derived monomers in photocurable thermosets and 3D printing resins, Schimpf et al. (2020) investigated the synthesis of limonene dimethacrylate (LDMA) from LD and MA. This biobased alternative was chosen due to its higher sustainability, solvent-free synthesis, and absence of estrogenic effects typically associated with bisphenol A glycidyl methacrylate (BisGMA), widely used in 3D printing and dental applications. The goal was to develop a less viscous resin to facilitate 3D printing by photopolymerization while retaining mechanical and thermal properties comparable to BisGMA. For this purpose, LDMA was synthesized by varying the molar ratio of MA/LO, reaction temperature, and catalyst type, also yielding oligomers derived from LDMA, as shown in Figure 6b. The study concluded that an excess of MA reduced resin viscosity but generated toxic residues, a limitation overcome by employing glycidyl methacrylate (GMA) and magnesium oxide (MgO), which acted respectively as a reactive diluent (forming glycerol dimethacrylate, GDMA) and nonvolatile salts. Furthermore, the application of LDMA in 3D printing was benchmarked against BisGMA, demonstrating comparable thermal and mechanical properties as well as effective processing into complex geometries, such as double-helix structures (Figure 6c).^87^

## 6 Thiol–Ene Polymerization

The reaction involved in this process is classified as a “click” reaction due to its high yield and minimal or negligible formation of byproducts.^2^ The advantages of the thiol–ene reaction include a high reaction rate, oxygen tolerance, and the possibility of obtaining highly cross-linked products when multifunctional thiols are employed.^4^ The thiol–ene reaction proceeds via a two-step radical mechanism consisting of repetitive chain propagation and transfer. Typically, the process requires an initiator, which may be thermal, such as AIBN, or light-activated, such as the photoinitiator 2,2-dimethoxy-2-phenylacetophenone (DMPA).^2,3^

The reaction begins with hydrogen transfer from the thiol to a primary radical, generated from initiator cleavage, resulting in an electrophilic thiyl radical (RS*) (Figure 7a, route i). The two most common types of thermal initiators are peroxides and azo compounds (e.g., AIBN), while acetophenone derivatives such as DMPA are the most common photoinitiators. Their cleavage pathways are depicted in Figure 7b.^5^ The thiyl radical then reversibly adds to one of the limonene double bonds (Figure 7a, route ii), forming an intermediate radical (RC*), which can easily abstract an electron-poor hydrogen from a thiol (S–H bond) (Figure 7a, route iii). In this final step, the thiyl radical is regenerated by chain transfer, yielding a stable thioether (C–S bond). Since this mechanism is repetitive and cyclic, the regenerated thiyl radical can re-enter the initial step of the reaction, which ceases only upon exhaustion of limonene double bonds or by recombination of thiyl radicals, as represented in route iv of Figure 7a.

**Figure 7:**
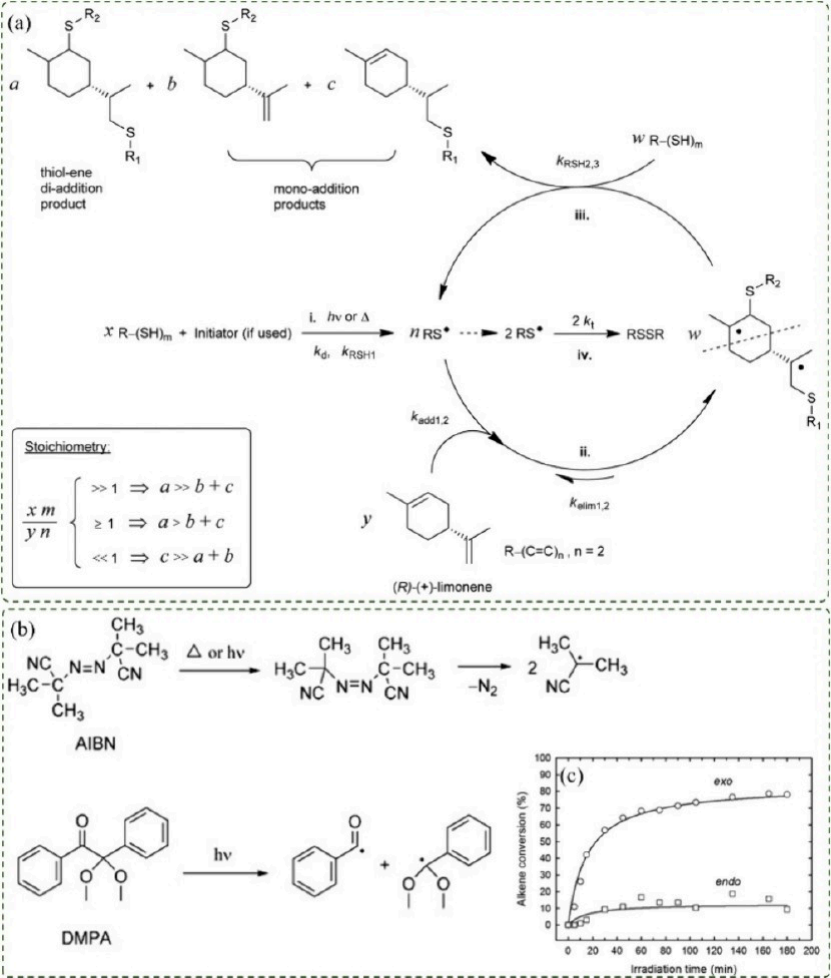
(a) Stepwise thiol–ene reaction scheme of limonene double bonds, adapted from ref 3. Available under a CC-BY 3.0 license. Copyright 2014 Claudino et al. (b) Decomposition of AIBN and DMPA initiators, adapted with permission.^5^ Copyright 2017 John Wiley and Sons. (c) NMR conversion profiles of *exo* and *endo* alkene functional groups as a function of time for a 1:0.5 thiol–ene mixture based on d-limonene, adapted from ref 2. Available under a CC-BY 3.0 license. Copyright 2013 Claudino et al.

The products of this reaction can be monoadducts, when propagation occurs via cleavage of only one of the limonene double bonds, or diadducts, when both double bonds participate in propagation. The ratio of these products depends on the thiol–ene stoichiometry. Figure 7a schematically summarizes how stoichiometry affects the reaction where *x* is the thiol ratio, *m* is the number of SH functional groups, *y* is the limonene ratio, and *n* is the number of limonene double bonds. Excess thiol favors the formation of diadduct polymers, while excess limonene favors more linear, monoadduct polymers. In general, thiol–ene polymerization yields translucent polymers with attractive physical properties for applications such as organic coatings, due to their strong adhesion to various substrates.^2,4^

### 6.1 Reactions with Bifunctional Thiols

Drozdov et al. (2019) compared the thiol–ene polymerization of siloxane-modified limonene and unmodified limonene with dithiols (ethanedithiol, butanedithiol, and hexanedithiol), aiming at the synthesis of oligomers to be employed as “*pre-*polymers” for further applications. The thiol–ene polymerization was carried out under UV irradiation at 365 nm, under solvent-free conditions, using benzophenone as the photoinitiator. All synthesized prepolymers were obtained as viscous, translucent, and yellowish liquids. The comparison focused on evaluating the effect of siloxane incorporation into the backbone and the influence of the methylene spacer length. The authors observed that an increase in the number of carbon atoms within the siloxane segments led to higher molar mass and dispersity, attributed to the formation of cyclic products during thiol–ene polyaddition, resulting from the enhanced mobility of siloxane monomers. For *pre-*polymers synthesized exclusively from limonene and dithiols, higher molar masses were achieved when longer-chain dithiols were employed, which was explained by the greater availability of reactive terminal groups in the thiol–ene polymerization. The thermal stability of the synthesized oligomers increased with longer methylene spacers, while the presence of siloxane had no significant effect on the degradation temperature. Furthermore, the incorporation of siloxane groups and longer methylene spacers reduced the glass transition temperature (*T*_g_) of the prepolymers. Regarding intrinsic viscosity, a significant increase was observed with longer methylene spacers, whereas siloxane groups showed no measurable influence on this property.^88^

Tarablsi et al. (2015) synthesized linear polymers using four dienic terpenes, including limonene, through reaction with ethylene glycol dithiol (EGDT) and the photoinitiator 1-[4-(2-hydroxyethoxy)­phenyl]-2-hydroxy-2-methyl-1-propane-1-one (I2959). This process was conducted under mini-emulsion conditions and compared with solution and bulk polymerizations, as the authors hypothesized that mini-emulsions could enhance both reactivity and molar mass of the products. They reported that the main challenge in obtaining high-molar-mass polymers lies in achieving stoichiometric balance between thiol and ene groups, which can be hindered either by the purity of the monomers or by local compositional fluctuations within emulsion droplets since EGDT is more soluble in water than terpenes. The reaction between limonene and EGDT achieved 98% conversion, yielding a poly­(thioether) insoluble in tetrahydrofuran (THF) and only slightly soluble in deuterated chloroform (CDCl_3_), which was attributed to the formation of crystalline domains along the polymer backbone. Overall, the study demonstrated the feasibility of employing mini-emulsion techniques for the photopolymerization of limonene with EGDT, with significant advantages over bulk and solution polymerizations, particularly in achieving high monomer conversions (above 95%).^89^

### 6.2 Reactions with Multifunctional Thiols

Claudino et al. (2013) reported the photopolymerization of limonene with both a monofunctional and a trifunctional thiol, namely, iso-tridecyl 3-mercaptopropionate (C13MP) and tris­(3-mercaptopropionate) (TMPMP), respectively, using DMPA as the photoinitiator. The aim of this study was to elucidate the thiol–ene coupling mechanism of limonene in CDCl_3_ solution, seeking to establish a relationship between alkene structure and reactivity. The authors observed that the coupling reaction at the exocyclic double bond of limonene proceeds 6.5 times faster than that at the endocyclic one, a trend confirmed by NMR analyses of the reaction between limonene and C13MP (Figure 7c), which revealed substantially higher conversion of the exocyclic bond compared to the endocyclic bond. Additionally, kinetic parameters such as the initiator decomposition rate were determined and subsequently applied in numerical simulations. The kinetic behavior was well described by a mechanistic model of thiol–ene polymerization, and the authors proposed that the higher reactivity of the exocyclic double bond arises from the relatively lower energy of the intermediate radical and the steric hindrance effects that kinetically regulate the insertion of thiyl radicals into the two different double bonds.^2^

Subsequently, Claudino et al. (2014) employed the trifunctional TMPMP and the tetrafunctional pentaerythritol tetra­(mercaptopropionate) (PETMP) in combination with limonene under both thermally initiated polymerization, using AIBN at 70 °C, and photopolymerization, using DMPA at 365 nm. Their goal was to maximize the cross-linking density of the resulting thermosets by introducing PETMP. Experimentally, it was observed that an excess of limonene relative to thiols favored monoaddition at the exocyclic double bond of limonene, while an excess of thiol, in a 1:0.5 thiol–ene stoichiometric ratio, promoted diaddition at both the exocyclic and endocyclic double bonds. Although the former pathway was kinetically more favorable for generating macromonomers, it hampered the removal of unreacted limonene due to its low vapor pressure; consequently, the latter pathway was selected, despite yielding highly branched, high-molar-mass oligomers alongside the desired macromonomers. After solvent evaporation, viscous, yellowish-ceria-scented liquid resins were obtained and subsequently combined with thiols to prepare limonene-derived films with varying stoichiometric ratios. The authors concluded that stoichiometric tuning of thiol and ene functionalities enables the preparation of resins with distinct architectures and tunable thermal properties.^4^

Claudino et al. (2014) further advanced these studies by investigating the application of C13MP and TMPMP in thiol–ene polymerization with limonene. In this work, the authors compared thermal and photochemical systems using monofunctional thiol C13MP to obtain kinetic data for numerical modeling by computer simulations. Once again, stoichiometric manipulations revealed that increasing the relative concentration of limonene enhances the selectivity of coupling at the exocyclic double bond. However, it was also established that the selectivity cannot be solely controlled by adjusting the initial reagent composition. For systems involving multifunctional thiols, simulations predicted that a stoichiometric excess of thiol yields oligomers with higher molar masses. Comparing temperature and light-induced systems, the authors concluded that photoinitiated systems exhibited higher reaction rates and lower incidence of side reactions, confirming that this methodology is suitable for industrial-scale synthesis processes employing limonene and multifunctional thiols.^3^

### 6.3 Synthesis of Thermosets and Additive Manufacturing Applications

In addition to the direct application of limonene, several of its derivatives have also been employed in thiol–ene reactions to obtain polymers for use as curing agents and thermosetting materials. Among these derivatives, epoxides, previously discussed in Section 4, can be applied in the synthesis of polycarbonates, polyesters, and polyethers. Acosta Ortiz et al. (2022) synthesized curing agents by combining a tetrafunctional thiol with a limonene-derived amine-functionalized intermediate, aiming to incorporate this curing agent into a biobased commercial epoxy resin to produce thermosets. The resulting curing agent contained tertiary amines, which reacted with epoxy groups to generate alkoxides that subsequently attacked other epoxides, initiating an anionic ring-opening polymerization (AROP) and forming polyethers. Simultaneously, the unsaturated moieties of the curing agent reacted with thiol groups to produce poly­(thioethers). These two polymers formed interpenetrating polyether–poly­(thioether) networks, whose rigidity varied depending on the relative proportion of poly­(thioethers) in the structure.^90^

In the studies of Li et al. (2017), high-performance thiol–ene networks were developed from PLimC. For this purpose, the authors first carried out the copolymerization of limonene oxide (LO) and CO_2_ using a zinc catalyst, yielding high-molar-mass PLimCs, which were subsequently reduced via transcarbonation reactions. The PLimCs were then cured with multifunctional thiols and thermal initiators through thiol–ene reactions involving residual unsaturation of the side groups. The resulting thiol–ene networks exhibited glass transition temperatures above 100 °C, together with favorable mechanical properties and high transparency.^42^ Continuing their work, Li et al. (2021) applied UV-curing thiol–ene reactions to LO/CO_2_-derived PLimCs, producing powder coatings with good durability and chemical resistance.^91^

3D printing has also been employed as a tool for the development of new limonene-based constructs. For instance, Constant et al. (2022) synthesized a copolymer from limonene and β-myrcene for use in 3D printing via digital light processing (DLP), as illustrated in Figure 8a. The resulting poly­(limonene-*co*-myrcene) was subsequently combined with a tetrafunctional thiol to obtain a printable resin. Through photo-cross-linking during 3D printing, the authors produced porous structures exhibiting shape-memory behavior. Figure 8b depicts the percentage of recovery to the original shape of the printed material over time, following deformation and subsequent heating.^49^ Similarly, Brooks et al. (2023) employed the same 3D printing technique. In their approach, copolyesters were first synthesized through the ring-opening copolymerization of limonene oxide and cyclic anhydrides. These polyesters were then combined with eugenyl methacrylate and PETMP (Figure 8c), resulting in mechanically stable three-dimensional objects. All printed materials displayed shape-memory effects upon heating (Figure 8d).^11^

**Figure 8:**
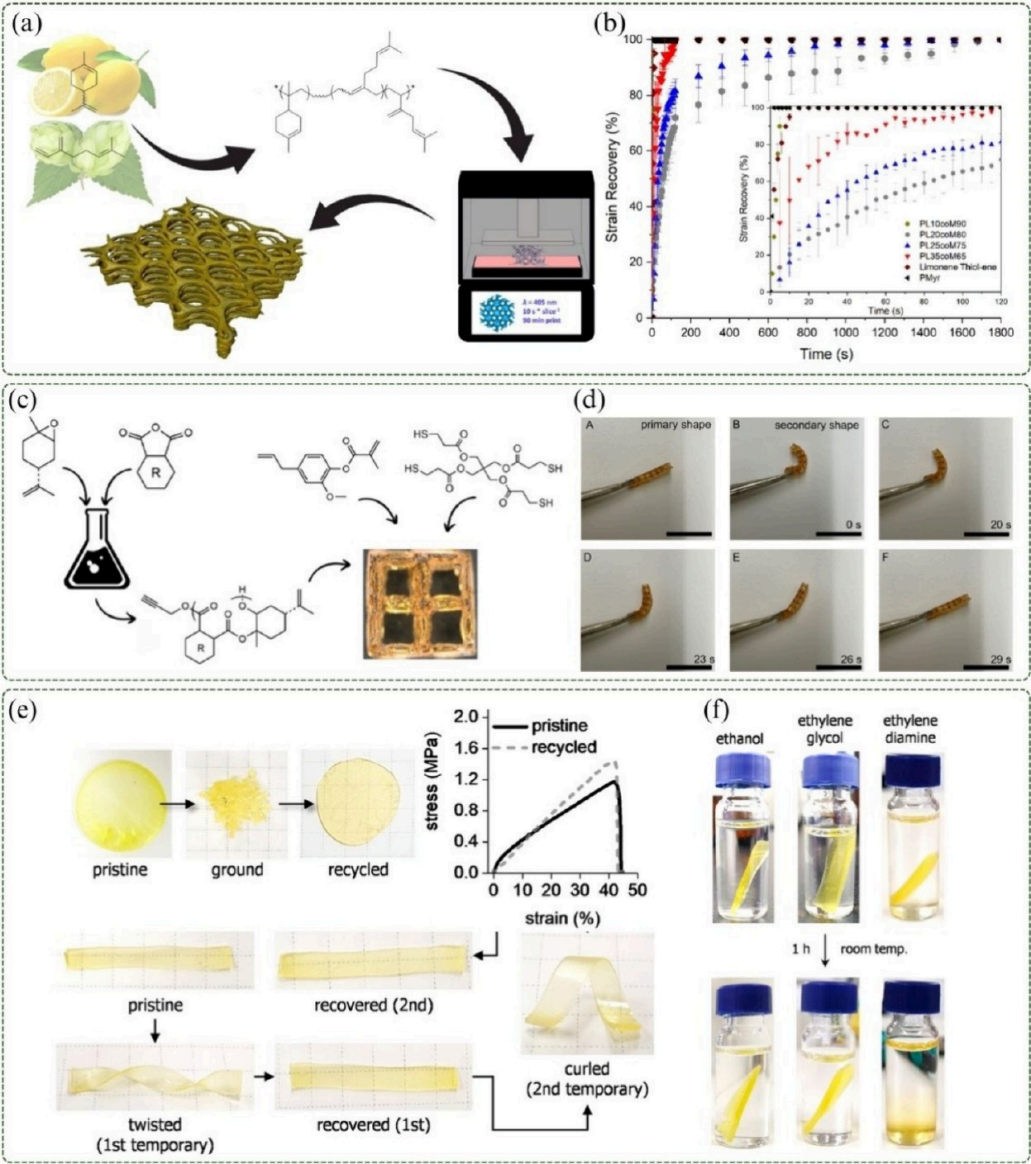
(a) Schematic of the application of limonene/β-myrcene copolymers for 3D printing, adapted with permission.^49^ Copyright 2022 American Chemical Society. (b) Deformation recovery of limonene/β-myrcene copolymer films at their *T*_gs_, adapted with permission.^49^ Copyright 2022 American Chemical Society. (c) Schematic of ring-opening polymerization of limonene for 4D printing applications, adapted with permission.^11^ Copyright 2023 American Chemical Society. (d) Deformation and shape recovery of photopolymers after 30 s at 160 °C, adapted with permission.^11^ Copyright 2023 American Chemical Society. (e) Recyclable thermoset and shape-memory behavior of limonene-based thermosets, adapted with permission.^10^ Copyright 2023 American Chemical Society. (f) Degradation of limonene thermosets in nucleophilic solvents, adapted with permission.^10^ Copyright 2023 American Chemical Society.

Taken together, these studies illustrate the breadth of polymerization strategies that have been explored to valorize limonene and its derivatives, each offering distinct advantages in terms of molecular control, achievable properties, and application scope while also exhibiting intrinsic limitations associated with monomer structure, reaction mechanism, and process conditions. In order to facilitate a clearer comparison between these diverse approaches and to guide the rational selection of polymerization routes depending on targeted material properties, processing requirements, and end-use applications, Table 3 provides a comparative overview of the main polymerization strategies reported for limonene-derived monomers, highlighting their key benefits, constraints, and typical domains of applicability.

**Table 3:** Comparative Overview of Polymerization Routes for Limonene-Derived Monomers

polymerization route	limonene-derived monomers best suited	key advantages	main limitations	when this route is most appropriate
**free-radical polymerization (FRP, controlled FRP, co/terpolymerization)**	limonene as a comonomer with acrylates, methacrylates, styrenics; cross-linked networks	broad methodological toolbox; simple initiators and processing; enables chemical functionalization and renewable content introduction into conventional radical polymers	strong allylic chain transfer reactions limit molecular weight and control; dispersity often broad; limonene mainly acts as a functional modifier rather than an efficient propagating monomer; mitigation strategies only partially effective	best suited when limonene is used to introduce functionality or biocontent into established radical polymer matrices, or in cross-linked systems where high *M*_n_ is not critical
**ionic polymerization (cationic/anionic)**	terpenes and limonene derivatives; limonene mainly in copolymerization	potential for improved structural control and selectivity; access to architectures difficult to obtain via radical routes	application to limonene remains limited and highly condition-dependent; sensitive to impurities; narrower operational window; still less mature than ROCOP-based strategies	appropriate for structure-driven material design where precise control is prioritized over robustness and scalability
**coordination polymerization (Ziegler–Natta, metallocenes, postmetallocenes)**	limonene or other terpenes as co- or ter-monomers in olefin polymerizations	industrially established platform; control over tacticity, crystallinity, and mechanical performance; efficient for polyolefin-based elastomers	limonene incorporation is typically low and mainly modifies properties; catalyst compatibility and feed purity are critical; limited access to limonene-rich polymers	relevant when targeting olefinic elastomers or modified polyolefins, with limonene acting as a renewable performance modifier
**ROCOP/ROP-based approaches (LO/CO**_**2**_ **, LO/anhydrides)**	limonene oxide (LO) generates poly(limonene carbonate) and/or related polyesters	most mature route for limonene-based polymers; yields well-defined polymers with high *T*_g_, narrow dispersity, and competitive performance; valorizes CO_2_; broad compositional tunability	requires specific catalysts, often elevated CO_2_ pressure; sensitive to hydroxyl impurities; techno-economic factors (catalyst cost, pressure, scalability) must be addressed	preferred route when high-performance, well-defined limonene-derived polymers are required, particularly PLimC-type materials with clear application potential
**thiol–ene (“click”) polymerization**	limonene and multifunctional thiols; networks, coatings, functional materials	high efficiency, oxygen tolerance, fast kinetics; excellent for cross-linked and functional materials; compatible with photocuring; enables dynamic or recyclable network designs	architecture strongly depends on stoichiometry; predominantly yields thermosets; reprocessability depends on network chemistry	ideal for coatings, photocurable systems, and advanced functional networks, where rapid curing and functional density outweigh linear chain requirements

## 7 Techno-Economic Feasibility and Circularity Strategies

A comprehensive perspective on the techno-economic feasibility and circularity of limonene-derived polymers was provided by Zhang et al. through an integrated life cycle assessment and process design study focused on the industrial production of poly­(limonene carbonate) from biomass-derived limonene and carbon dioxide. In this work, conceptual process flowsheets were developed and simulated at an industrially relevant scale, enabling the simultaneous evaluation of environmental impacts, energy integration potentials, and production costs. The authors demonstrated that the estimated cost of poly­(limonene carbonate) (US$ 1.36–1.51 kg^–1^, depending on feedstock origin) is comparable to that of fossil-based polystyrene, highlighting the economic competitiveness of limonene-based polycarbonates under optimized conditions. Importantly, the study identified upstream processing steps, particularly limonene oxidation using *tert*-butyl hydroperoxide, as the dominant contributors to both environmental burden and operational cost, outweighing the impact of the CO_2_/epoxide copolymerization itself, which proceeds under relatively mild pressure and temperature conditions. By comparing limonene sourced from citrus waste and engineered microalgae and by incorporating energy recovery from residual biomass, the authors further showed that carbon-negative production scenarios are achievable when polymer synthesis is coupled with appropriate waste valorization strategies. Overall, this analysis establishes a process-level framework in which economic viability and circularity are governed primarily by feedstock integration and upstream chemistry rather than by polymerization efficiency alone.^92^

Building on these process-oriented insights, subsequent studies have addressed techno-economic feasibility from the complementary perspective of material processability and end-of-life performance, which ultimately determine the practical deployment of limonene-based polymers. Neumann et al. demonstrated that the limited melt processability of poly­(limonene carbonate), arising from its high melt viscosity and narrow processing window, can be effectively mitigated through compounding with biobased additives such as ethyl oleate. This approach enabled melt processing without thermal degradation while preserving optical transparency and mechanical integrity and, importantly, allowed multiple reprocessing cycles without significant loss of properties, representing a meaningful advance toward practical recyclability.^93^ In a related architectural strategy, Carrodeguas et al. reported block polycarbonate-polyester systems derived from limonene oxide, carbon dioxide, and ε-decalactone that combine enhanced toughness and elasticity with selective, catalyzed depolymerization to the original monomers, thereby directly linking improved performance with chemical recycling pathways.^94^

More recently, the focus has shifted toward application-driven material design, explicitly targeting the requirements of industrial processing and product integration. Höferth et al. addressed the brittleness and processing limitations of poly­(limonene carbonate) through the development of graft copolymer architectures with tailored graft density, resulting in materials with significantly improved ductility, toughness, and compatibility with conventional processing techniques. By demonstrating their use as toughening agents, compatibilizers, and electrospinnable materials, this work positions poly­(limonene carbonate) closer to real-world applications traditionally dominated by fossil-based plastics, while maintaining its biobased origin and recyclability potential.^95^ Complementary process-level considerations were further explored by Belinchón et al., who combined ionic-liquid-catalyzed limonene carbonate synthesis with catalyst recovery strategies and process simulation. Their analysis reaffirmed that, despite efficient CO_2_ utilization during polymerization, upstream steps such as limonene extraction and epoxidation remain decisive contributors to environmental and economic performance, reinforcing the need for integrated, circular process design to enable scalable and sustainable limonene-based polymer production.^96^

While the studies discussed above primarily focus on thermoplastic limonene-derived systems, circularity considerations are equally critical for thermosetting materials, which are traditionally associated with limited recyclability. To address this limitation, Choi et al. (2023) designed a recyclable and degradable limonene-based thermoset. To assess recyclability, the authors prepared thermoset disks, cut them into small pieces, and reprocessed them in a press at 150 °C for 1 h. The reprocessed films exhibited mechanical properties comparable to the original thermoset, as shown in Figure 8e. In this study, a multifunctional thiol was employed to form a highly branched limonene polymer, which was subsequently cross-linked with a Meldrum’s acid derivative. This cross-linking produced reversible covalent bonds, enabling polymer degradation in nucleophilic solvents such as ethylenediamine (Figure 8f), generating reusable products suitable for the synthesis of biobased polyureas. The resulting polymer also exhibited shape-memory properties, demonstrated by its torsion and recovery at 30 °C (Figure 8c).^10^

## 8 Conclusions and Future Prospects

Due to its unique structure and broad availability from renewable sources, limonene stands out as one of the most promising monoterpenes for the synthesis of next-generation polymeric materials. Its chemical versatility, evidenced by the presence of conjugated double bonds and chiral centers, enables not only selective epoxidation reactions but also the generation of derivatives such as oxides, lactams, and acrylated monomers, which serve as versatile platforms for a wide range of polymerizations. Throughout this review, it has become evident that limonene polymerization pathways, whether radical, ionic, coordination-based, or “click” processes such as thiol–ene coupling, have advanced considerably in recent years, enabling the synthesis of polyesters, polyethers, polycarbonates, and polyamides with tunable properties and high potential for applications in packaging, biomaterials, coatings, and additive manufacturing.

Progress in the copolymerization of limonene oxide with anhydrides or carbon dioxide, for example, demonstrates not only the feasibility of CO_2_ capture in highly transparent and robust polycarbonates but also the construction of block copolymers and terpolymers with tunable thermal and mechanical properties. Likewise, studies on the thiol–ene functionalization of limonene highlight innovative pathways for the development of thermosetting resins and 3D-printed materials with shape-memory behavior and chemical recyclability. Recent investigations into limonene-derived lactams and their subsequent transformation into high-performance polyamides further point toward opportunities to replace fossil-based polymers in structural and engineering applications.

Nonetheless, several challenges remain to be addressed, spanning both molecular-level control and process-level implementation. From a polymer chemistry perspective, further advances are required in the precise control of macromolecular dispersity and in the mechanistic understanding of propagation and chain transfer events associated with limonene’s allylic double bonds. At the same time, recent techno-economic and life cycle assessments indicate that practical constraints, including catalyst selection and recovery, monomer purification, upstream oxidation chemistry, and overall process integration, play a decisive role in determining the industrial viability of limonene-based polymers, particularly for ring-opening (co)­polymerization routes involving carbon dioxide. In this context, poly­(limonene carbonate) emerges as the most mature and extensively investigated limonene-derived polymer to date, benefiting from a comparatively robust body of literature encompassing catalyst development, structure–property relationships, processability, recyclability, and life cycle performance. As such, it represents a benchmark system for assessing both the opportunities and limitations associated with the large-scale deployment of limonene-based polymers.

In parallel, the incorporation of circularity-oriented strategies has emerged as a critical requirement for the long-term implementation of these materials. Approaches such as chemical recyclability through selective depolymerization, the design of reprocessable or dynamically cross-linked networks, and solvent- or catalyst-assisted recovery pathways provide viable routes to align high-performance materials with circular economy principles. Future research is therefore expected to focus not only on improving catalyst efficiency and selectivity but also on reducing environmental burdens associated with upstream transformations, enhancing tolerance to renewable feedstock impurities, integrating biofabrication routes for limonene production from agro-industrial residues, and developing continuous and scalable processes. Taken together, these advances consolidate limonene not merely as a high-value biobased molecule but as a strategic platform for renewable polymer production that coherently integrates material performance, techno-economic feasibility, and circularity.
